# Genomic variance in Yucatan pigs and detection of donor-derived cell-free DNA after heart transplantation

**DOI:** 10.1371/journal.pone.0334235

**Published:** 2025-10-27

**Authors:** Michelle Mendiola Pla, Joseph A. Prinz, Kishen Mitra, Franklin H. Lee, Matthew F. Smith, Alejandro Alvarez Lobo, Carmelo A. Milano, Dawn E. Bowles, Devjanee Swain-Lenz

**Affiliations:** 1 Division of Cardiothoracic Surgery, Duke University Medical Center, Durham, NC,; 2 Sequencing and Genomics Technologies Core Facility, School of Medicine, Duke University, Durham, NC,; 3 Division of Surgical Sciences, Duke University Medical Center, Durham, NC,; 4 Department of Molecular Genetics and Microbiology, School of Medicine, Duke University, Durham, North Carolina; Tokyo Women's Medical University, JAPAN

## Abstract

Acute rejection, one of the most devastating complications that can occur following organ transplantation, is caused by antigenic differences between the organ donor and the recipient. Following cardiac transplantation, an estimated 12% of patients will experience at least one episode of moderate or severe acute rejection in the first year after transplantation. To better understand the genetic mechanisms underlying acute rejection, Yucatan pigs (YP) serve as an ideal preclinical model. Translatability of the YP preclinical model relies on the fidelity observed between preclinical and clinical pathologies. Cell-free DNA (cfDNA) analysis has emerged as a blood-based, non-invasive screening tool for acute rejection of solid organs following transplantation. We present a detailed characterization of the genomic variance in YPs. The degree of variance matches that observed in humans, enabling for the ability to detect and distinguish between donor-derived and recipient-derived fragments isolated from the transplant recipient’s blood.

## Introduction

Despite outcomes improving after cardiac transplantation over the last 30 years, acute rejection (AR) remains a major complication that confers significant morbidity to patients. An estimated 12.6% of patients experience at least one episode of moderate or severe AR within the first year after transplantation. Experiencing even one episode of AR has been described as a main determinant for increased morbidity [1,2]. Preclinical disease models provide a platform to characterize human disease pathophysiology and test potential therapeutic interventions prior to use in humans. Crucial aspects of a reliable preclinical model are having defined methods and parameters that are translatable and clinically relevant when testing novel advanced therapies [3,4].

Yucatan pigs (YP) are being used more frequently for preclinical studies given their genetic and immunologic similarities to humans [5–7]. Additionally, this breed has a slower growth rate and overall smaller size in comparison to other pig breeds. The final size of an adult YP heart is approximately the same size as an adult human heart. Because of these reasons, YP hearts are being used more frequently for translational research applications, as well as for clinical use in xenotransplantation. The swine leukocyte antigen (SLA) closely matches the antigenicity of its human homolog, human leukocyte antigens (HLA). Mismatch of either SLA or HLA between a donor and a recipient is a major cause for the development of AR. Through complete mismatching of SLA between YPs with a limited peri-operative period of immunosuppression, we have demonstrated that it is possible to induce the development of AR in a controlled and consistent manner that results in fulminant AR within a consistent and reproducible timeframe [8,9].

While YPs are an ideal model for investigating AR it remains challenging to assess for AR, particularly in an intra-abdominal heterotopic heart transplantation model [10]. The current clinical gold standard for monitoring AR is histologic assessment of serial endomyocardial biopsies (EMB). In humans these biopsies are obtained under local anesthesia and performed transvenously using a bioptome under ultrasound guidance where right ventricular septal myocardium is obtained and assessed for presence of leukocyte infiltration and myocardial damage characteristic of acute rejection. However, this technique presents a safety risk to perform in a porcine heterotopic, intra-abdominal heart transplantation model as serious bleeding complications can arise from inadvertent perforation of the transplanted heart that can compromise the health of the individual YP or negatively affect experimental results requiring the use of general anesthesia and endotracheal intubation [11].

**Fig 1:**
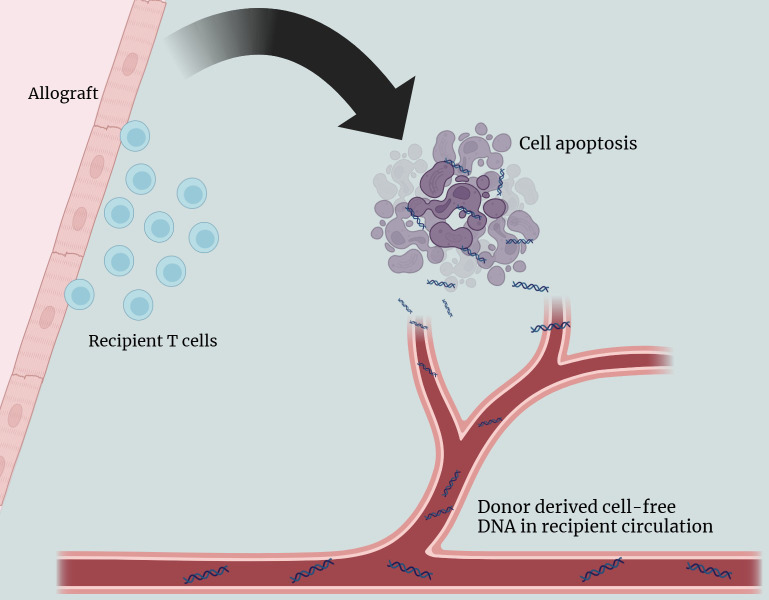
Association of acute rejection and release of donor derived cell-free DNA in recipient circulation.

Analysis of cfDNA has been growing in popularity for monitoring AR after cardiac transplantation given that it has minimal associated morbidity [12,13]. In clinical applications, researchers have successfully used the analysis of cell-free DNA (cfDNA), but an analogous method has yet to be described in YP. Furthermore, donor-derived cfDNA (ddcfDNA) analysis is a non-invasive blood test that can detect subacute AR earlier than EMB histologic grading analysis [14,15]. When cells from the allograft undergo apoptosis as in the context of AR, they release short DNA fragments into the recipient’s circulation (Fig 1) [16]. The use of donor-derived cfDNA (ddcfDNA) as a marker for AR screening is based on its association with increased apoptosis during AR progression in allografts. This success of this assay has led to the development of multiple commercially available platforms to quantify ddcfDNA for AR screening across various organ transplants [17].

Recipient T cells target donor allograft cells which leads to the progression of apoptosis. This process releases small fragments of DNA from the allograft cells that enter the circulation of the recipient. These DNA fragments can be detected and identified as donor derived, indicating that acute rejection occurring.

An understanding of the genomic variance that exists within the YP genome would allow for the development of a sensitive cfDNA assay that could detect AR just as reliably as has been achieved clinically. Here we characterize variance within the YP genome using whole genome sequencing (WGS) and single-nucleotide polymorphism (SNP) analysis to determine the feasibility of being able to detect and quantify ddcfDNA in the recipient pig’s circulation following intra-abdominal heterotopic heart transplantation.

## Materials and Methods

### Animals

The care and treatment of pigs followed the *Position of the American Heart Association on Research Animal Use* [18]. This study was approved by the Duke University Institutional Animal Care and Use Committee (A122-22–06). Female Yucatan pigs (Sinclair Bio Resources, Auxvasse, MO) aged 7–9 months were used. The weight of the pigs during the study ranged from 13–42 kg. All pigs underwent swine leukocyte antigen (SLA) haplotyping and blood-typing prior to selections [10,19–21]. Transplant pairs were chosen that were blood-type compatible and fully SLA Class 1 and Class 2 mismatched to induce AR.

### Cardiac transplantation and acute rejection monitoring

Assigned pairs underwent intra-abdominal heterotopic cardiac transplantation as described in Mendiola Pla *et al* [10]. Prior to all surgeries, the animals were sedated and underwent endotracheal mechanical ventilation for the entirety of the procedure. General anesthesia using ketamine (5–33 mg/kg) and midazolam (0.2–0.5 mg/kg) intravenously was used then sedation was maintained using isoflurane gas (1–3%). Buprenorphine (0.005–0.01 mg/kg) was administered intramuscularly post-operatively for analgesia management. Donor hearts were procured in standard fashion and underwent normothermic *ex vivo* perfusion using a TransMedics Organ Care System^^®^^ (Andover, MA) device for 2 hours. The hearts were then implanted heterotopically into the recipient pig’s abdomen. To induce AR in a controlled manner, the recipient pigs received immunosuppression therapy for 2-weeks post-operatively. For immunosuppression, the recipient pigs began receiving tacrolimus intramuscular injections three days prior to transplantation with a trough goal of 5–15ng/L. Following transplantation, the animals continued receiving tacrolimus injections. They were also started on a methylprednisolone taper and mycophenolate mofetil 400 mg twice daily. The immunosuppression was stopped post-operative day (POD) 14 [9]. Animal health and behavior were monitored two to three times a day during the experimental period by trained personnel. Animals were then closely monitored for the progression of AR where cardiac activity was assessed daily by palpation and graded on a 0–4 + scale, where 0 corresponds to no palpable pulse whereas 4 + corresponds to a vigorous pulse that can be seen through the skin. Troponin blood levels and echocardiography were assessed weekly throughout the survival period.

On post-operative day (POD) 30, the pigs underwent EMB of their transplanted heart to monitor for histologic evidence of AR through hematoxylin and eosin (H&E) staining, also as described in Mendiola Pla *et al* [11]. Animals were survived until there was fulminant graft rejection, defined as complete cessation of allograft activity, or if allograft survival surpassed greater than 100-days. Animals were euthanized within 24-hours of determining rejection or within 1-week of the allograft surviving past 100-days. Humane endpoints were defined for euthanasia such that if an animal was found to be moribund with altered mentation or injury a veterinarian was consulted for intervention recommendations and decision to proceed with possible euthanasia. For euthanasia, animals again underwent endotracheal mechanical ventilation and sedation as described above. To procure the hearts, a sternotomy and laparotomy were performed and the hearts were each arrested using Del Nido cardioplegia infused directly into the respective aortic root of each heart. Each heart was subsequently excised from the animal.

### Genomic and cell free DNA sequencing

**Fig 2:**
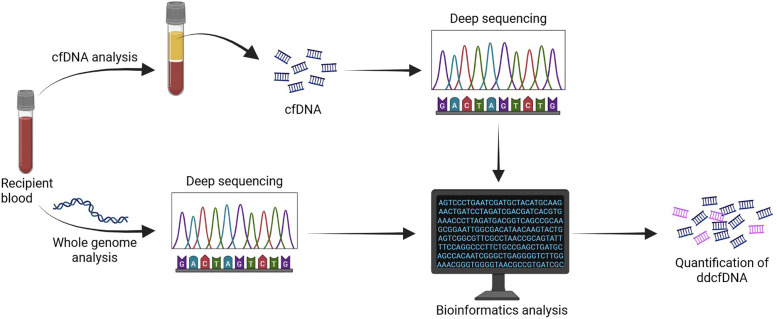
Schematic of whole genome and cell-free DNA analysis.

Whole blood samples were collected intravenously from pigs pre-operatively and at the time of euthanasia. They were flash frozen prior to storage at −80°C. Genomic DNA (gDNA) was isolated using the ReliaPrep Blood gDNA Miniprep System (Promega Corp., Madison, WI). Purified genomic DNA samples were stored at −20°C prior to use for WGS. Plasma was isolated from whole blood samples following centrifugation at 850 x g for 10 minutes to separate red blood cells. These were also flash frozen prior to storage at −80°C. Cell free DNA (cfDNA) was isolated using the QIAamp DSP Circulating NA Kit (QIAGEN, Hilden, Germany). Purified cfDNA samples were stored at −20°C prior to use for sequencing (Fig 2).

Whole blood samples were collected from all the pigs (n = 12) at baseline. Genomic DNA was isolated from this and was used for whole genome analysis. Whole blood samples were again collected from the recipient pigs (n = 6) at the endpoint for allograft acute rejection. Plasma was isolated from the whole blood and cell-free DNA was subsequently isolated. The cell-free DNA was then sequenced, matched, and quantified using bioinformatics analysis and was identified as either donor-derived or recipient-derived.

The Duke Sequencing and Genomics Technologies Core Facility performed WGS. Average cfDNA fragment size was calculated using the Agilent Bioanalyzer 2100. DNA samples were sheered to approximately 500 base pairs (bp) and used to build DNA libraries with the KAPA HyperPrep kit (Roche catalog: 07962363001). Whole genome libraries were pooled to equimolar concentration and sequenced on the Illumina NovaSeq 6000 to produce 150 bp paired-end reads. cfDNA libraries were pooled to equimolar concentration and sequence on the Illumina NextSeq 1000 to produce 150 bp paired-end reads.

### Sequencing analyses and variant calling

Sequencing data was processed using fastp to trim low-quality bases and Illumina adapters from the 3’ end of reads [22]. Reads were then aligned to the Sscrofa11.1 version of the porcine genome with the BWA algorithm [23]. PCR duplicates were flagged using the PICARD Tools software suite (http://broadinstitute.github.io/picard/). Alignment processing and variant calling were performed using the GATK toolkit following the Broad Institute’s Best Practices Workflow [24,25]. Prior to variant calling, each read’s quality scores were refined using BaseRecalibrator, genotypes were determined by HaplotypeCaller per-sample after which samples-sets were merged using GenotypeGVCFs. Once variants were called, the following filters were used to determine and exclude potential false-positives using the authors’ recommended “hard-filter” strategy: SNPs having QD < 2.0 or QUAL < 30.0 or SOR > 3.0 or FS > 60.0 or MQ < 40.0 or MQRankSum < −12.5 or ReadPosRankSum < −8.0; INDELs having, QD < 2.0 or QUAL < 30.0 or FS > 200.0 or ReadPosRankSum < −20.0. Genotypes for the whole-blood and cell-free datasets were processed separately. As a negative control, we mapped reads to the Y chromosome, which <0.01% of the chromosome was detected. We compared our final list of SNPs to dbSNP database [26].

### Identifying donor-derived DNA among cell-free DNA

Two methods, a phased and an unphased method, were used to calculate the percentage of ddcfDNA in circulating cfDNA isolated from recipients. This was done to ensure the accuracy of identifying ddcfDNA in recipient circulating blood. The phased method used WhatsHap5 to create phased haplotypes from WGS followed by identification of donor-derived SNPs using vcfeval to assign a SNP as either donor- or recipient-derived. Following this, vcfeval was used again to compare each set to the corresponding cell-free genotypes and the number of genotypes found within the cfDNA.

The unphased method used the GATK pipeline to identify SNPs from WGS to create an index to sort genotypes in cfDNA to either the donor or the recipient. A simple index was used to determine if a given genotype was unique to one individual or shared between the donor and respective recipient. Similarly, genotypes that were called in the cell-free sample of the corresponding recipient with at least five reads of support were compared to the sets determined above. This method was used to interrogate if the depth of coverage at single SNPs was sufficient to identify ddcfDNA.

### Statistical analysis

Statistical analyses were performed using R Statistical Software version 4.3.1 (Vienna, Austria). A p-value of <0.05 was considered significant. Pearson correlation test was used to assess for linear correlation between two variables. Results are presented as Pearson’s correlation coefficient (*r*) and range from −1 to +1. Paired, single-tailed Student’s t-test was used to compare continuous variables with normal distribution. Statistical notations used in the figures: p > 0.05, not significant (ns); p < 0.05, *; p < 0.01, **; p < 0.001, ***; p < 0.0001, ****.

## Results

### Whole genome sequencing of Yucatan Pigs

A median of 625M reads were sequenced for a medium depth of 31x coverage for each individual animal (range 26x – 35x) (S1 Table). 13,277,787 SNPs and 3,137,076 indels were identified, of which 2,313,327 and 2,378,099 were novel variants not assigned in dbSNP, respectively. Of note, the variants reported in the dbSNP database are derived from several diverse pig breeds. To describe the distribution of unique SNPs across the YP genome, a strict filter was applied on the variants above and analyzed 1,057,314 high confidence variants, of which 845,414 were SNPs and 211,900 were indels. For each base of the whole genome sequences between individuals from donor-recipients, a SNP was characterized as either shared or distinct between individuals. The deep sequencing demonstrated an average of 3.42 (SD = 0.068) SNPs per kilobase. Importantly, genetic diversity was distributed across the YP genome and not exclusively at the SLA region of chromosome 7. This level of genome-wide genetic divergence in 12 YP supports that genome-wide cfDNA analyses could be useful as a marker of AR in a YP model of organ transplantation.

### Donor-derived cell-free DNA detection after acute rejection

**Fig 3:**
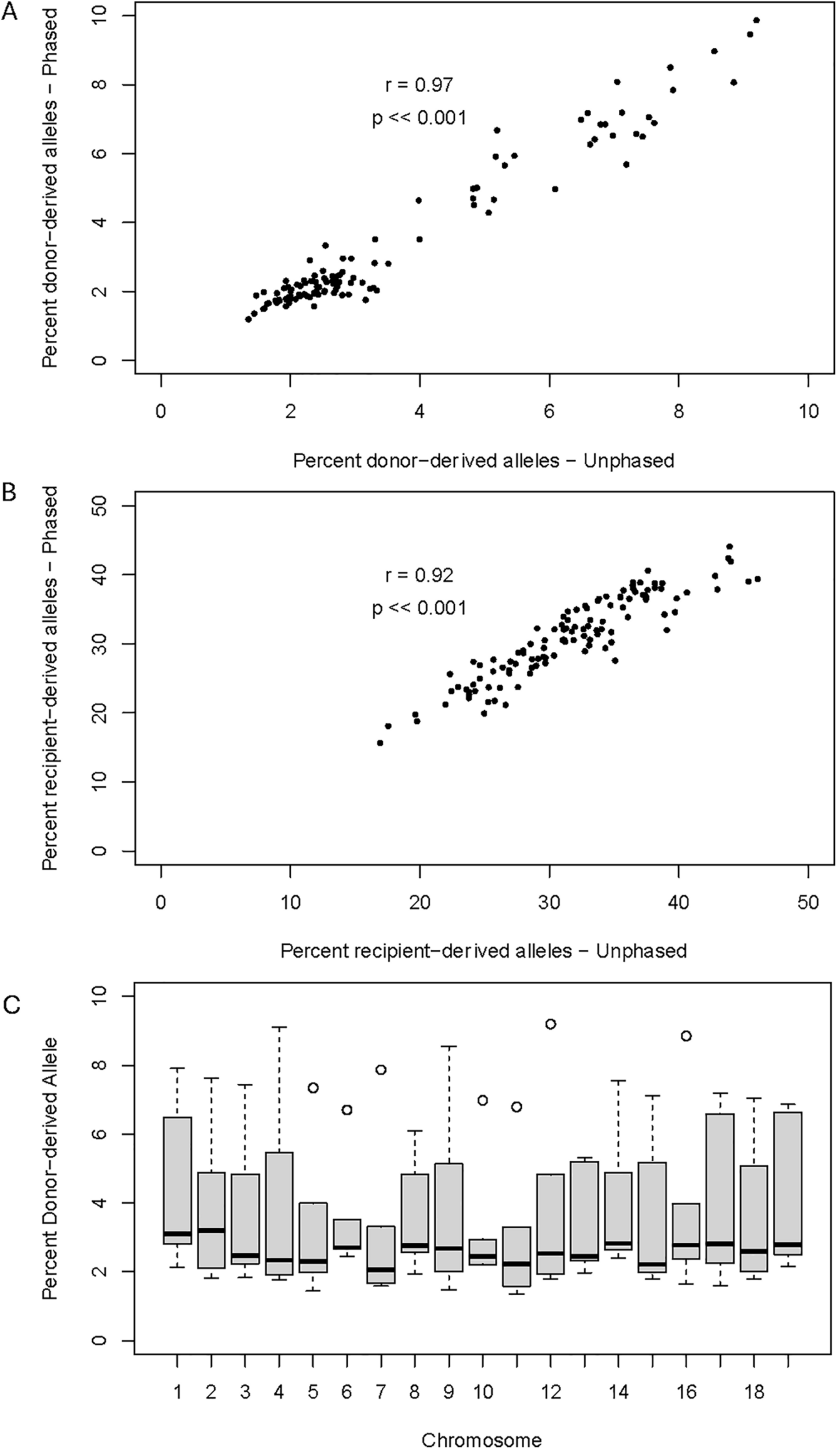
Donor-derived cell-free DNA detection at acute rejection.

5 YPs experienced fulminant AR within the allotted study timeframe, while 1 YP did not (Table 1). All YPs were included in the analysis. For both the phased and unphased haplotype method, we analyzed 7.7–10.8 M SNPs that were unique between a given donor-recipient pair (S2 and S3 Tables). ddcfDNA was able to be identified in the recipient’s circulating blood using either analytic method. Detectable ddcfDNA percentage ranged from 1.8–7.2% and 1.8–7.4% of reads using the phased and unphased method, respectively (Fig 3A). Both methods had high correlation of percent donor-derived variants (*r* = 0.97, p < 0.001) and percent recipient-derived variants (r = 0.92, p < 0.001), indicating that distinguishing ddcfDNA is feasible and accurate with or without phasing analysis (Fig 3B). Additionally, we observed that alleles detected on circulating ddcfDNA mapped in a comparable distribution to all of the YP chromosomes, suggesting that ddcfDNA fragments derive from throughout the YP genome with comparable frequency (Fig 3C)(S2 and S3 Tables).

**Table 1:** Donor-derived alleles in cell free DNA genotyping.

Sample	Troponin (ng/L)	Time to rejection (Days)	Baseline fragment size (bp)	Endpoint fragment size (bp)	%dd-cfDNA phased	%dd-cfDNA unphased
A	434	41	2319	3887	2.20	2.55
B	1458	89	2167	4258	1.77	1.95
C	11185	33	2283	5493	7.23	7.44
D	1144	53	3463	4014	2.21	2.51
E	24601	39	2701	4587	4.89	4.80
F	526	Did not reach (sacrificed day 106)	2690	3797	2.01	2.22

ddcfDNA was detected whether using a phased or unphased analytic method at the time of AR. (A) When measuring ddcfDNA, there was significant correlation between the phased and unphased measurements. Each measurement shown is the percent ddcfDNA at one chromosome. (B) When measuring recipient-derived cfDNA, there was significant correlation between the phased and unphased measurements. Each measurement shown is the percent recipient cfDNA at one chromosome. (C) Boxplot representation of percent ddcfDNA measured across each chromosome. Median and 95% C.I. are shown.

### Donor-derived cell-free DNA increases with severity of rejection

**Fig 4:**
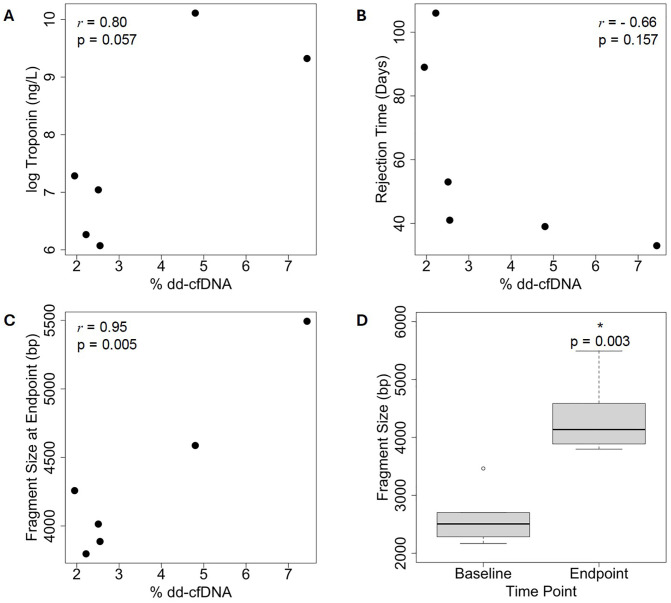
Donor-derived cell-free DNA correlates with acute rejection measurements.

In the clinic, percent ddcfDNA is used as a biological marker of impending AR. Therefore, we asked whether percent ddcfDNA correlated with standard markers and rate of progression of AR. In conjunction with measuring ddcfDNA from recipient blood samples, troponin was also measured from these samples as a marker of myocardial injury secondary to acute rejection. We asked whether ddcfDNA correlated with troponin levels (Table 1, Fig 4A). Percent ddcfDNA correlated moderately with log-transformed troponin levels. However, the correlation did not reach statistical significance (r = 0.80, p = 0.057) most likely due to our small sample size. We also measured the Pearson correlation between percent ddcfDNA and the rate of progression of AR (Table 1, Fig 4B). Percent ddcfDNA weakly and negatively correlates with a faster rate of AR (r = −0.66, p = 0.157). While not a standard marker of AR, cfDNA fragment size increases during apoptosis. Notably, the average size of cfDNA fragments measured between 40−10,000 bp were larger than expected in recipient samples at AR (Table 1, Fig 4C). We asked whether percentage ddcfDNA correlated with larger fragment size at the time of AR endpoint (r = 0.95, p = 0.004). We also asked if cfDNA increased in size from baseline to endpoint, and find that fragment size is significantly larger at the AR endpoint compared to baseline (p = 0.003) (Fig 4D).

Dot plots of percent ddcfDNA (x-axis) and (A) log transformed troponin (ng/L), (B) rejection time (Days), and (C) fragment size at endpoint (y-axes). Pearson’s correlations (*r*) and corresponding p-values are displayed. (D) Box plot comparing average cfDNA fragment size at baseline (prior to transplantation) versus at the time of study endpoint show a significantly larger fragment size at the study endpoint (paired single tail Student’s t-test).

## Discussion

### Yucatan pigs have genetic variation comparable to humans

We present an exploratory analysis demonstrating the feasibility of sequencing cfDNA to detect ddcfDNA in a porcine model of AR following heart transplantation. We used clinically relevant methods to quantify the percentage of ddcfDNA measurable within the transplant recipient’s blood [13]. We began our approach towards developing a ddcfDNA assay in YPs by using WGS and bioinformatics analysis to determine if there was sufficient genetic variation between individual pigs to be able to distinguish between donor-derived cfDNA alleles and recipient-derived alleles. WGS analysis revealed nucleotide divergence of approximately 0.1% between individual YPs. This amount of variation is consistent with the genetic diversity that is observed between human individuals [27–29]. Not only does this validate the ability to distinguish between donor-derived and recipient-derived cfDNA fragments, it also supports the utility of YPs as preclinical models for translational research. Furthermore, the identification of 13 million SNPs across the YP genome with approximately 2.3 million novel SNPs aligns with the magnitude of identified SNPs in recent studies. This highlights the potential for designing targeted genomic panels to enhance the sensitivity and specificity of ddcfDNA assays for AR monitoring in YPs [30,31].

### Cell-free DNA phenotypes as biological markers of acute rejection

Quantification of ddcfDNA has been growing in clinical use as an alternative diagnostic modality for monitoring allografts post-transplantation for detection of AR [12,13,32–34]. The rationale for its use as a marker for screening for AR comes from the association between the increasing rate of cell apoptosis and the progression of AR. cfDNA analysis has grown in popularity because it is a non-invasive test simply requiring a blood sample from the patient, and for its ability to detect subacute AR even prior to AR detection by EMB. Following our analysis of YP genomic variance, we proceeded to analyze all of the cfDNA isolated from YPs at the time of euthanasia for fulminant AR. We chose to focus on this timepoint to validate the feasibility of our method to detect ddcfDNA in the presence of AR from the sharp tissue biopsies. All of the animals, except for Animal F, progressed to fulminant AR. Histologic grading demonstrated severe AR in all of the allografts except for the allograft in Animal F which only had scattered areas of mild to moderate AR.

We were able to distinguish ddcfDNA fragments from recipient cfDNA fragments circulating in the recipient’s blood. Most of the blood samples contained levels of ddcfDNA that were comparable to levels reported in clinical studies that were highly correlated with AR [12]. Interestingly, Animals C and E had much higher levels of ddcfDNA detected, approximately 4.8% and 7.4% respectively. These two animals also had higher levels of troponin and the largest cfDNA fragments detectable within the cohort possibly indicating a higher degree of myocardial injury. Conversely, we detected high levels of circulating ddcfDNA in Animal F. We believe this was possibly due to the presence of subacute AR that was yet to manifest as clinically detectable AR given the finding of scattered areas of mild to moderate AR.

Another interesting finding from this study was that genetic variation within the YP genome was observed genome-wide and not isolated exclusively to the SLA region on chromosome 7 despite the breed having a highly conserved ancestral lineage. These variable loci could prove to yield candidate regions for developing high-sensitivity genotyping probes to screen for AR through more rapid and economical benchtop assays. An additional future direction beyond this study would be to identify a ddcfDNA percentage threshold to diagnose AR in a YP following heart transplantation, similar to what has been described clinically.

## Conclusion

The use of YPs in translational research offers unique advantages. Their genetic similarity to humans enables the development and validation of biomarkers, such as size and quantification of ddcfDNA, which are crucial for non-invasive monitoring of graft health. Additionally, the controlled genetic background of YPs minimizes confounding variables, facilitating focused investigations into rejection mechanisms and therapeutic interventions. By bridging preclinical models with clinical applications, YPs play a pivotal role in advancing the understanding and management of allograft rejection, ultimately driving innovations in cardiac transplantation care.

## Supporting information

S1 Table Sequencing table. (XLSX)

S2 Table Unphased donor-derived cell-free DNA identification. (XLSX)

S3 Table Phased donor-derived cell-free DNA identification. (XLSX)
